# Servicewomen’s experiences of managing pelvic health in occupational settings

**DOI:** 10.1177/17455057231183839

**Published:** 2023-06-28

**Authors:** Kate Freire, Simone O’Shea, Rod Pope, Rob Orr

**Affiliations:** 1 Three Rivers Department of Rural Health, Charles Sturt University, Wagga Wagga, NSW, Australia; 2 Charles Sturt University, Albury, NSW, Australia; 3 Bond University, Gold Coast, QLD, Australia

**Keywords:** female, genitourinary, military personnel, occupational health, pelvic floor

## Abstract

**Background:**

Despite increasing numbers of women serving in defence forces worldwide, little is currently known about how servicewomen manage their pelvic health in the traditionally male environment of the military.

**Objectives:**

The aim of this study was to explore the impacts of pelvic health issues on Australian Defence Force servicewomen and their experiences of managing their pelvic health in occupational settings.

**Design:**

A qualitative hermeneutic design.

**Methods:**

Telephone interviews were conducted on six currently serving female members of the Australian Defence Force located Australia-wide. A semi-structured interview guide, based on the study objectives, was used to guide the audio-recorded interviews. Data were analysed thematically.

**Results:**

Nine themes were identified. The first six themes explored the experiences of servicewomen in maintaining their pelvic health, including suppressing the urge to go, adjusting hydration depending on toilet access, managing menstruation, regaining ‘full’ fitness postpartum, awareness and prevention of pelvic health conditions, and inhibiting conversations about women’s health. The last three themes explored how servicewomen coped with pelvic health conditions, including self-managing symptoms, diagnosing and treating pelvic conditions, and support for servicewomen’s pelvic health.

**Conclusion:**

This study suggests workplace culture, low levels of insight into pelvic health norms, and limited healthcare strategies within the Australian Defence Force to support female pelvic health have contributed to servicewomen self-managing pelvic health issues using approaches that may have had significant impacts on their health and well-being.

## Introduction

Pelvic health encompasses normal functioning and management of the bladder, bowel, and reproductive organs, and of the surrounding structures, such as muscles and ligaments.^1^ Women are more likely than men to experience several conditions affecting pelvic health, such as incontinence, urinary tract infections, and voiding issues.^1,2^ Prevalence of pelvic health conditions among women varies across the lifespan, with a recent study reporting that one in five women will experience ongoing impacts to their bladder health.^3^ Women encounter disadvantage through health inequities, including the trivializing of women’s complaints,^4^ poor access to services, low health literacy, and sociocultural discrimination, such as unequal power relationships between men and women.^5^ In addition, the acknowledged history of gender bias in health research has meant that health issues that predominantly affect women have received significantly less attention and funding, than health issues that predominantly affect men.^4^ The socioeconomic impacts are substantial, impacting women’s quality of life, their relationships, and financial costs on individuals and society for treatments and products for managing these conditions.^6^ Economic analysis suggests that prioritizing women’s health would lead to improved overall population health and enhanced productivity through greater engagement and retention of women in the workforce.^7^

In 2012, the Australian Defence Force (ADF) identified increasing the numbers of women serving as a priority.^8^ Recent survey data have shown an increase in the proportion of the workforce that is female, from 11.8% in 1991 to 18.1% in 2019.^9^ The growing female representation in the ADF, and other defence forces worldwide, necessitates a focus on areas of health where men and women differ, such as pelvic health, to ensure appropriate prevention strategies and healthcare are provided.^10^

Previous studies investigating the pelvic health of servicewomen have been predominantly conducted in the United States. In 2016, a systematic review of US servicewomen’s genitourinary and reproductive health identified a lack of healthcare providers with training in women’s health, particularly in deployment settings,^11^ suggesting that defence forces are not able to respond to servicewomen’s health needs. This was corroborated in another recent review that identified multiple barriers for servicewomen to consult healthcare providers about menstruation suppression or regulation.^12^ Barriers included health literacy of both servicewomen and healthcare providers, limited availability of female healthcare providers, and cultural factors in US Defence Forces, including stigma for seeking care.^12^ Yet, like in the general population, pelvic health issues are common among servicewomen, with up to one-third of US servicewomen having been affected by genitourinary infections and urinary incontinence.^13,14^ In addition, a 10-year-old, qualitative study, which explored how US servicewomen managed their pelvic health when deployed, identified three key themes.^15^ The *deployment setting* theme described their work context, including the physical context, encompassing elements, such as their heavy, tight-fitting uniforms, the availability and variety of female products in military stores, social issues, such as privacy, and the job requirements. *Dynamics of trust* explored how servicewomen relied on themselves and interacted with others (co-workers, family, healthcare providers, leadership) to access information, supplies, and support. And finally, *sphere of control* examined the activities that servicewomen employed to maintain and manage their genitourinary conditions while on deployment.^15^ These studies have focused on US servicewomen during deployment, but it should be noted that not all service personnel will deploy during their career in the ADF.^16^

Building on this background of research on servicewomen’s pelvic health in the US military context, in 2019, we commenced a mixed-methods programme of research to explore the pelvic health of Australian servicewomen. The programme of research began with a comprehensive survey of ADF women and female veterans.^16^ Overall, 491 servicewomen (currently serving and veterans) completed the survey, and their demographic profile was observed to be comparable to the demographic profile of servicewomen reported in the Defence Census 2019.^16^ The survey found that lower urinary tract symptoms were commonly experienced during service. For example, 27% of respondents reported regular symptoms of urinary incontinence during their service.^16^ The second part of the research programme, using a small number of respondents who volunteered after completing the survey, is described in this article. It was a qualitative study to explore the in-depth experiences of a small number of ADF women in relation to their pelvic health.

### Aims of the research

The aims of the current research were to explore (1) how female ADF personnel manage their pelvic health, (2) the impacts of pelvic health conditions on servicewomen, (3) servicewomen’s experiences in relation to the organizational support and services they had received in managing their pelvic health, and (4) what other assistance would be valued by servicewomen to manage their pelvic health.

## Methods

A qualitative hermeneutic methodology was used. The research was carried out between December 2019 and July 2020. Individual interviews were conducted via telephone because participants were located throughout Australia. Participants were encouraged to choose a quiet, private space for the interview; some took part from their work office, and others took part from home.

### Recruitment and participants

A convenience sampling method was used, with participants invited to take part in interviews after completion of an online survey on related topics.^16^ The survey invitation was distributed via print advertisements (ADF newspapers) and social media posts (Facebook). Adult biological females (sex assigned at birth), who had actively served in the ADF for at least 6 months, were eligible to participate. Exclusion criteria included individuals who did not identify as female from birth, were younger than 18 years of age, or had not served for longer than 6 months in the Australian Navy, Army, or Air Force. Survey respondents who indicated an interest in the interviews and supplied their email contact details for this purpose were contacted by a researcher (S.O.), informed about the research (via email correspondence), and invited to participate in the interviews. Following written consent from each participant, an interview time was arranged.

Six servicewomen and two veterans took part. This article focuses on the contemporary occupational context of ADF servicewomen by examining the data from the six currently serving ADF women. The data from veteran servicewomen will be presented in a separate paper to enable differentiation between current and historical experiences. Demographic information of the participants is provided in an aggregated form to prevent identification of individual participants and protect their privacy (Table 1).

**Table 1.** Aggregated demographic information of participants.

Ages	Average 43 years, range 20–63 years
Service arm	Navy, n = 2 Army, n = 4
Employment status	Full-time, n = 4 Part-time, n = 2
Current rank	Commissioned officer, n = 4 Warrant officer or non-commissioned officer, n = 1 Other rank, n = 1
Length of service	Average 22.4 years, range 2.33–38 years
Times deployed	Average no. 3.5, range 0–9
Categories of work roles^a^	Management, business, administration, or education role, n = 3 Aviation role, n = 1 Communications, IT, or intelligence role, n = 3 Combat or security role, n = 2 Healthcare, chaplaincy, or science role, n = 3 Logistics, hospitality, or other support role, n = 1

a Four servicewomen had been employed in more than one work category.

### Data collection and management

The semi-structured interviews were conducted by one female researcher (K.F.). The researcher was experienced in qualitative research and was a qualified physiotherapist. A topic guide (see Supplementary Material) was developed and specified the subject areas to be explored during interviews, based on a literature review, and input from the research team, which provided expertise in relevant fields. Congruent with a semi-structured approach, questions were not asked in a particular order but ordered flexibly according to the participant and their experiences. The researcher was cognisant of the personal nature of the topics under discussion and approached the topics with sensitivity and vigilance for the participant’s well-being. The researcher paraphrased responses back to the participants during the interviews to ensure she had understood the participants’ meaning and provide participants with opportunities to reflect and clarify their meaning. This was an important approach because the study employed a single interview methodology. The interviews varied in length between 45 and 90 min. All interviews were audio-recorded and transcribed verbatim. The transcripts were de-identified, and pseudonyms were assigned to each participant prior to analysis to protect their privacy.

### Analysis

Systematic thematic analysis was divided into several phases. Initial analysis occurred concurrently with data collection. Following each interview, the researcher wrote a summary of the key interview points. These summaries were compared, by members of the research team, after each new interview to identify preliminary themes or topics, recognize when no new topics emerged from an interview, and judge when themes had been explored in-depth (data saturation). Each transcript was then coded according to the research aims. Where possible, the researcher chose codes which used the participant’s language to maintain a link with the participant’s voice. Codes were then refined by identifying similar codes across all transcripts, ordering them into code trees and combining excerpts from the six transcripts into one transcript based on coding. For example, the initial coding tree relating to bladder continence included frequency, change of clothes, access to toilet, awareness of toilet locations, and maintaining hydration. Codes were refined into themes and subthemes by identifying commonalities and differences in responses across the data. During this phase, several reflexivity strategies were used to limit the impact of researcher bias, as one researcher performed the coding described. Critical feedback throughout the analysis phase was sought from the research team, and interpretation of the themes was tested by writing about them, ensuring each aspect was supported by participants’ excerpts.

## Results

The results are divided into two sections. The first section explores the experiences of the ADF servicewomen in maintaining their pelvic health while in their roles as service personnel and includes six themes: suppressing the urge to go, adjusting hydration depending on toilet access, managing menstruation, regaining ‘full’ fitness postpartum, awareness and prevention of pelvic health conditions, and inhibiting conversations about women’s health. The second section explores how servicewomen cope with pelvic health conditions as service personnel and includes three themes: self-managing symptoms, diagnosing and treating pelvic conditions, and support for servicewomen’s pelvic health.

### Maintaining pelvic health as service personnel

Additional excerpts are provided as support for each theme in Table 2.

**Table 2.** Maintaining pelvic health as service personnel: interview excerpts illustrating themes.

Themes	Excerpts
Suppressing the urge to go	The requirement to be on watch without the ability to take a break to go to the toilet exists. For a minimum of four hours, well any length of time really, but a normal watch at sea was four hours long. So, you went to the toilet before you went on watch and you didn’t leave that bridge, that ops room for another four hours. That is quite a normal working cycle when you are at sea. In times of higher tempo that time would increase. The requirement would be six or eight hours of a working day. (Blake)
Adjusting hydration depending on toilet access	Quite often you try to not drink a lot, so you get a bit dehydrated because there is very few facilities for females to use out bush. You know if you are a bloke, you can go behind a tree, with a female it is not quite so easy. And quite often you are in limited numbers during those periods. So, it might be ten males for one female. So, you try and hold on until you cannot hold on any longer. (Cameron)
Managing menstruation	It is difficult especially if you have your periods to keep clean. So quite often that’s hard to, you know if you use tampons you have got to try and bury things. So, you don’t have bathing facilities in most cases. So, most females I know, we tend to take ‘wet ones’ and things like that. And try and have a bit of a bird bath. (Cameron) It’s a pretty common thing, especially with girls in defence, when we do field activities that are particularly strenuous, the difficult stuff, most girls will either go one of two streams. Their periods either stop entirely, or as soon as their body goes under stress, they will get their periods regardless of whether they are in their cycle. (Alex) When you come back, and you haven’t had a period for a while you quite often have a really heavy one if you have been taking a series of pills. And so, you can have quite severe pelvic pain and heavy bleeds. So that makes it really difficult to go to work. (Cameron)
Regaining ‘full’ fitness postpartum	There is a lot of pressure from people to upgrade. . . . And personally, I think that probably the whole thing should be ‘oh you have delivered, here is your pelvic floor physiotherapist. (Drew)
Awareness and prevention of pelvic health conditions	Sometimes it baffles me how we, as women, how little we know about our own body. (Blake) If you are running a PT session involving putting a pack on your back . . . as the person in charge, I don’t think that person would be thinking, oh I’ve got ten females in this, I just have got to make them aware that if they have any issues with their pelvic floor afterwards, get on to it straight away. Whereas it is more a case of, when we finish this, if you have got problems with your knees, or your feet are sore, . . . go straight down to the RAP [regimental aid post]. (Jamie) We are now starting to have a few more midwives and people like that who can give a lot of education. (Cameron)
Inhibiting conversations about women’s health	Like I am talking when I first joined, it wasn’t really accepted that you had female problems. You just had to suck it up and get on with it. The attitudes have changed. (Cameron) The sheer numbers of women who are entering the system mean that perhaps some areas that we were hesitant to discuss, you know even just maybe getting your period at sea, how to manage that. (Blake) We tend to gloss over some of the troubles that you have from a pelvic health perspective, women in Defence. Because it is such a male dominated workforce you generally don’t discuss those things either around workplaces because they are dominantly male. (Alex) But I mean the thing is that you can’t just go, I am a girl. And we don’t want to do that either. The attitudes, a lot of them are self-imposed. I think that is because we just do. It’s really hard to explain. We just do it. . . . We don’t want to be seen as a girl. . . . I think the other thing is the attitudes are, I mean the culture is still you have got to be your best and you have got to do your best. (Drew)

Names used in the results, including tables, are pseudonyms.

#### Suppressing the urge to go

Due to the nature of their work, suppressing the urge to relieve themselves when their bladder was full was described by participants. Blake’s excerpt in Table 2 described how her role in the Royal Australian Navy meant that she could not access the toilet for at least 4 h when on watch. She emphasized that this was a legal requirement of her role, thus something that all service personnel who undertook this role understood, irrespective of their biological sex, and abided by when performing this role.

#### Adjusting hydration depending on toilet access

Service personnel reported being aware of the importance of adequate fluid consumption. Jamie said, ‘Pretty much we are big on plugging [advocating for], drink your fluids . . . Be hydrated, make sure that you have got your water bottle with you’. They advised this had been part of service culture for several decades and something they endeavoured to follow.

However, access to toilets was sometimes limited or unhygienic; or there was a lack of privacy for servicewomen in certain work contexts, such as out bush [on a field exercise], on deployment, or when performing specific roles or tasks (Table 2). In these circumstances, servicewomen would drink less, risking dehydration; and resist urges to relieve themselves because they were uncomfortable doing so in an exposed and male-dominated environment.

Thus, maintaining good levels of hydration for servicewomen was more complex than simply adhering to the instruction to drink adequate amounts of fluid because they must also weigh up the consequences of maintaining adequate hydration in contexts where toilet access was limited. Time for ablutions, privacy, and access to clean bathroom facilities were also factors identified by servicewomen when managing their menstruation.

#### Managing menstruation

Servicewomen described difficulties while managing menstrual periods out bush. For example, Cameron (Table 2) explained that the service members could be out bush for several weeks and due to factors identified in the last section, servicewomen sometimes kept their tampons in longer than they should, which could result in infections (and the potentially life-threatening condition, toxic shock syndrome).^9^

Servicewomen reported changes to their menstrual cycle during times of intense activity (Table 2). They reported that many of them used hormonal contraception, partly to gain some control of the timing of their menstruation and reduce the likelihood of dealing with the added difficulties of menstruation during field activities and deployment. However, using contraception to miss several cycles of menstruation at inconvenient times could subsequently lead to heavy and painful periods for some servicewomen (Table 2). This could impact their work and increase their concern about leakage through clothing, especially among those who wore white navy uniforms.

#### Regaining ‘full’ fitness postpartum

Servicewomen recognized the importance of fitness to perform their roles in the ADF and that they were responsible for their own return to fitness postpartum while on maternity leave. However, there was also a consensus that the ADF emphasis on return to work was not holistic but focused on regaining aspects of fitness required to pass fitness tests. There was a perception of pressure to upgrade faster than required, with less acknowledgement of, or support for other aspects of return to health postpartum (Table 2). Servicewomen emphasized that regaining full fitness postpartum should also include checking up on the new mother’s pelvic health – a practice that, according to the participants’ experiences, was infrequently implemented in the ADF.

#### Awareness and prevention of pelvic health conditions

Servicewomen noted how little they knew about their pelvic health as young women prior to enlistment and how little they learned during their early years in the service (Table 2). They recounted how they used their experience as a service member and their evolving knowledge about pelvic health to help support and informally mentor other, usually newer, servicewomen. ‘I think you need to be aware of your own body. . . . I will quite often have a meeting with the females around how to manage their hygiene for instance, when they are out bush’ (Cameron).

Some participants expressed frustration that preventing issues with women’s pelvic health was still not part of regular management of the health of personnel in the ADF. In Table 2, Jamie contrasted physical training instructors’ (PTI) overt encouragement to seek prompt treatment for any musculoskeletal symptoms after physical training (PT) to their silence about pelvic floor and continence conditions.

However, others, such as Cameron (Table 2), had noticed an improvement in the number of personnel able to provide education and support to servicewomen during pregnancy and postpartum.

#### Inhibiting conversations about women’s health

Service members felt that attitudes towards women’s health in the ADF had improved during their time in service (Table 2). Cameron identified two reasons why she thought that attitudes of service personnel towards women in the ADF had become more accepting: personnel with negative attitudes to women serving were leaving the service, and the numbers of women serving in the ADF were increasing. Larger cohorts of servicewomen allowed for greater openness in discussions about women’s issues and their management during service. Blake also felt the cultural change within the ADF reflected a shift to a more open, mature, and practical approach to women’s health in the wider culture among younger generations in Australia. In contrast, other participants, such as Alex (Table 2), felt the male-dominated workplace stifled discussion about female pelvic health.

Servicewomen acknowledged the impacts of their own attitudes, and their group’s attitudes, towards both their service and their pelvic health. They identified two aspects of service culture that contributed towards servicewomen’s approaches to their pelvic health (Table 2). They described the approach of service personnel to their occupation and the ethos they adopted in doing ‘their best’, which led to a diligence in performing their roles irrespective of their own circumstances. And, as shown by Drew’s excerpt in Table 2, where Drew referred to ‘girl’ in a derogatory tone, servicewomen also perceived that to be a part of the team, servicewomen could not be seen to be a ‘girl’. Both these aspects highlight the effect of culture on women’s health issues and complexities for military personnel and defence forces in addressing women’s health issues.

Servicewomen explained that the culture of not being ‘seen as a girl’ stifled discussion on women’s health, which resulted in limited opportunities for finding out what normal was for their pelvic health. ‘I don’t think there is an opportunity to find out what that norm is. Or if there is even issues’ (Alex). Servicewomen’s lack of insight into what was normal, and the lack of information provided about women’s health in the ADF contributed to servicewomen self-managing significant pelvic health conditions before seeking help and treatment.

### Coping with pelvic health issues as a service member

#### Self-managing symptoms

Servicewomen reported putting up with and self-managing their symptoms and the impacts of those symptoms for significant periods of time because they did not understand what was normal. They described how living with pelvic health conditions, such as urinary incontinence or frequency, could interfere significantly with their daily lives, including their work. ‘Walking was not a problem at all. But anything more than that and I was instantly leaking. And coughing, sneezing, it was depressing. It was debilitating from an activity perspective’ (Blake). Overwhelmingly, the interviewees described their embarrassment of living with their conditions. They coped with them using several strategies to manage their symptoms in the workplace, which have been summarized in Table 3.

**Table 3.** Strategies employed by servicewomen to self-manage pelvic health issues.

General strategies • Monitored and restricted fluid intake • Awareness of location of toilets – priority in unfamiliar contexts • Used sanitary pads – to reduce risk of leakage when unable to access toilet • Access to changes of clothes • Limited their physical activity levels
Strategies used specifically on deployment • Bucket overnight in room to relieve themselves – for urgency and frequency symptoms • Gauging timing of bathroom breaks to allow extra time to undress and handle equipment safely, particularly when wearing personal protective equipment, such as body armour, and carrying equipment, such as rifles • Used sanitary pads – but difficult to access on deployment • Washing clothes in bucket when on deployment due to limited laundry facilities

#### Diagnosing and treating pelvic conditions

Participants described how the limited pelvic health prevention measures in their work contexts were mostly directed towards cancer screening, but they reported positive experiences with doctors in the ADF referring them promptly for specialist review when they finally consulted them, including to physiotherapists with a speciality in women’s health (Table 4). They pointed out, however, that some servicewomen had experienced challenges accessing physiotherapists with specific training in treatment of women’s health issues.

**Table 4.** Interview excerpts illustrating theme diagnosing and treating pelvic conditions.

I can’t complain about the assistance given to me. I could say to one of the doctors on base that I would like to see a specialist, and yep there is no problem. They won’t not refer you on. They are quite happy to. (Jamie) I know here in [name of base], there is not one physio wise on base, that has that training. So, the amount of time that I have been sent to physio in the past and spoken to a physio on base and they have basically thrown their arms in the air and said ‘well I can’t really help you. You need to see a physio specific to that area. You need to see them off base’. . . . But when we have to go outside Defence it can be a little bit hard to get to appointments. (Jamie) It was really only about 18 months ago . . . And seeing a surgeon, GP [general practitioner], and, subsequent to surgery, some physios, to a specialist in incontinence, that my military experience and having to hold on for lengthy periods of time in my 20’s started to become part of the conversation. (Blake)

Names used in the results, including tables, are pseudonyms.

A common theme, from servicewomen who had given birth, was that they had attributed their pelvic health conditions solely to this factor, and it was at specialist appointments that they gained insight into the possible impact their work demands may have had on their pelvic health conditions (Table 4).

#### Supporting servicewomen’s pelvic health in the ADF

Servicewomen had several suggestions about supporting women’s pelvic health in the ADF, starting with the development of greater awareness of conditions, such as bladder desensitization. ‘This is something for the young women at sea to be aware of. If these things can appear later on in life based on a lengthy time in your twenties, then that is an important conversation to have’ (Blake). They acknowledged that it was not an easy topic for service personnel to engage with. ‘I suppose you can put the information out there but again it is going to be up to people, the individuals that want to engage with that information. Because it is not an easy topic’ (Alex). They also pointed out that pelvic health education was important for all service personnel. ‘But by the same token some of that could be for men as well. Because doing weights and things like that there’s a reason that you wear a belt’ (Drew). A summary of the suggestions of interviewees to support servicewomen pelvic health is provided in Table 5.

**Table 5.** Participants’ suggestions for improving management of pelvic health issues.

Increase prevention education focusing on: • Commencing education about pelvic health for service personnel in basic training. • Education about pelvic health norms. • Education about the impact of ‘holding on with full bladder’ for development of bladder desensitization issues. Promote regular toilet breaks, ‘listening to your bladder’, at times when not on watch, for service personnel whose roles include being on watch. • Promote the effectiveness of simple strategies to maintain pelvic health: for example, pelvic floor exercises, going to toilet when bladder full. • Educate PTI to incorporate awareness of pelvic floor health and strength into musculoskeletal monitoring and encourage service personnel to seek help when needed.
Increase monitoring of pelvic symptoms and conditions: • Add to health monitoring questionnaires.
Promote usefulness and importance of specialist services to manage and treat pelvic conditions to encourage servicewomen to seek help earlier.

## Discussion

The increasing numbers of women serving in defence forces, both in Australia and internationally, necessitate greater understanding of how female military personnel currently manage their pelvic health and the impacts of pelvic health conditions on their service. The current study extends the findings from the study by Wilson and Nelson^15^ on US servicewomen’s experiences when on deployment to show that Australian servicewomen also experience occupational contexts when not on deployment that can impact their pelvic health. Key findings from this study included occupational contexts which restricted access to toilets and privacy, and cultural aspects of the workplace and servicewomen that limited opportunities to identify female pelvic health norms and led some servicewomen to self-manage significant pelvic health conditions prior to seeking treatment. Servicewomen reported that when they did consult, they found that doctors were keen to provide access to specialist care; subsequently, servicewomen were keen to share new insights into the management of pelvic health with other service personnel and informally mentored less experienced servicewomen.

Servicewomen reported they moderated their fluid consumption and manipulated their menstrual cycle when they worked in contexts where toilet access and privacy were limited. This finding concurs with the findings from a small number of other studies from the US military over the past 26 years^11^–^15^ and suggests that the impact of these factors on the pelvic health of servicewomen in military workplaces has not been a focus. The lack of focus is concerning because of the potentially serious risks associated with the ways servicewomen manage their menstrual cycle during service. For example, tampon misuse, from inadequate changes of tampons when out bush, put servicewomen at risk of menstrual toxic shock syndrome, a syndrome which may potentially result in multiple-organ failure and death.^17^ A recent 6-month case–control study found that women who reported wearing a tampon, during the day for more than 6 h, and during sleep for more than 8 h, have a two- and threefold increased risk, respectively, of developing menstrual toxic shock syndrome.^18^ Furthermore, the impact of some management strategies used by servicewomen may negatively affect their occupational performance, with potential to then impact the health and well-being of those around them, in addition to their own health and well-being. For example, limiting their fluid intake leading to possible dehydration. Dehydration has been found to increase the risk of heat illness and reduce alertness and ability to concentrate, leading to poorer performance in simple motor tasks.^19,20^

Servicewomen described occupational contexts where, due to operational requirements, service personnel were expected to work without access to toilets or time for breaks for 4 h or more. Delaying voiding can lead to bladder desensitization and health sequelae of pain, bladder storage and voiding issues, urinary tract infections, and increased risk of urinary incontinence.^21^ In non-military populations, a greater prevalence of bladder desensitization conditions is found in women compared to men.^2^ Servicewomen noted that the occupational contexts they described were universal to their role, irrespective of their sex. Although, we could find no data on bladder desensitization rates in servicemen, it appears a reasonable hypothesis that some servicemen may also be at risk of developing bladder desensitization from chronic, infrequent voiding practices while undertaking their duties and experience the subsequent health sequelae during or after their service. This hypothesis offers defence forces the opportunity to provide pelvic health education and promote norms about voiding and bladder hygiene, particularly when personnel are not under operational constraints. However, an inclusive approach should *not* be mistaken for a sex-neutral approach, as discussed in the next paragraph.

Servicewomen reported that they had limited understanding of their pelvic health prior to joining the ADF. Their work ethos meant that they completed their work irrespective of their own health circumstances and described how the predominantly male environment stifled opportunities to identify female-specific norms and discuss female pelvic health. The low level of understanding of female pelvic health and norms identified in the current study concurs with the findings of previous studies indicating that women had poor insight to the severity of their pelvic health condition at their first consultation with a specialist.^22,23^ Taken together, these findings suggest that women’s knowledge of pelvic health norms would benefit from greater public health messaging about female pelvic health throughout a woman’s lifespan and the impact of childbirth on pelvic health. However, public health messages alone are unlikely to significantly impact a servicewomen’s experience of addressing and managing their pelvic health; the workplace culture must also be addressed. Servicewomen described a male-dominated workplace culture where women felt they should deny their female (girl) identity in the workplace and not discuss women’s health issues in the workplace. Traditionally, male-dominated workplaces, such as defence forces and construction industries, have tended to address the increasing numbers of women entering those workplaces by denying the relevance of biological sex in the workplace and this has resulted in a silencing of women’s experiences.^24^ These authors suggest that such sex-neutral approaches have led the underlying primary (male) culture to prevail, hindering culture change, and resulting in psychological distress and attrition for all sexes, as traits, attitudes and actions traditionally recognized as female are disdained.^24^ Thus, acknowledging, promoting, and addressing servicewomen’s differing health needs may also indirectly benefit all service personnel by contributing to a workplace culture that acknowledges and accepts the differences between biological sexes.

In our study, ADF servicewomen found that, during consultation with health personnel, doctors were keen to provide access to specialist care. This finding diverges from previous studies from the United States, whereby military women did not feel they had adequate access to the gynaecological care they needed.^11^–^15^ However, these previous studies investigated the experiences of servicewomen on deployment, in contrast to the current study which explored servicewomen’s overall experiences in the ADF, including while on deployment. Thus, our findings may not indicate an improvement in women’s access to specialist gynaecological care while on deployment.

The servicewomen interviewed were solution-focused. They recalled how they mentored other, less experienced servicewomen, and suggested ideas to improve experiences of their fellow service personnel to maintain their pelvic health and manage pelvic health conditions in the ADF (Table 3). Practical suggestions from workers who have experience of managing their conditions under occupational constraints may provide invaluable insights into the pragmatic management of pelvic health conditions in the ADF.

Evaluation of preventive pelvic health education programmes, increased monitoring of pelvic health via health screening to enable timely treatment, and ways of increasing numbers of female health professionals with specialist knowledge in women’s health to ensure prompt and easy to access follow-up care are all critical avenues for future research. Future research should consider participatory approaches that include servicewomen, as the end-users, as this would allow health research to use current servicewomen’s insights in informing practical solutions in the unique and complex defence context. It should be noted that these avenues will necessarily shift focus away from descriptive studies, investigating the prevalence of conditions, to timely intervention studies that aim to improve the experiences, health, and well-being of current, and future, servicewomen.

### Limitations of study

This study was conducted using a small number of servicewomen and this, together with the qualitative approach of the research, limits the generalizability of the findings. The qualitative approach employed was suitable for exploratory research, and useful insights were gained into the current issues that servicewomen have encountered, and practical management suggestions they proposed; but it is acknowledged that other servicewomen are likely to give differently nuanced results. It is unknown whether experiences of specific minority groups are represented in this study’s findings and future research would be warranted to explore whether experiences of such groups within the ADF differ from those reported here.

## Conclusion

The findings of this study suggest the work ethos of servicewomen and their low levels of insight into pelvic health norms, together with the workplace culture (including the predominantly male context) and limited healthcare strategies within the ADF to support management of female pelvic health issues, have contributed to servicewomen self-managing pelvic health issues and using approaches that may have had significant and potentially very serious impacts on their health and well-being. Servicewomen identified several practical suggestions to highlight and improve management of pelvic health within the evolving culture in the ADF, including increased monitoring and education. Specific education suggestions included developing greater awareness of the impacts of bladder desensitization, which can result from operational demands restricting toilet access for 4 h or more. Finally, our findings suggest that limited access to hygienic and private toilet facilities is still an issue for servicewomen out bush and on deployment and may lead to servicewomen limiting their fluid intake or not relieving themselves when appropriate resulting in possible health sequelae.

## Supplemental Material

sj-docx-1-whe-10.1177_17455057231183839 – Supplemental material for Servicewomen’s experiences of managing pelvic health in occupational settings Click here for additional data file. Supplemental material, sj-docx-1-whe-10.1177_17455057231183839 for Servicewomen’s experiences of managing pelvic health in occupational settings by Kate Freire, Simone O’Shea, Rod Pope and Rob Orr in Women’s Health

## Declarations

**Ethics approval and consent to participate:** Approvals for the study were gained from the Human Research Ethics Committees of the Australian Departments of Defence and Veterans’ Affairs (099-19), Charles Sturt University (H19271), and Bond University (TC01733).

**Consent to participate:** Written consent was obtained prior to interview being arranged. At interview, the interviewer informed the participant of the interview process and gained verbal consent before proceeding with the interview.

**Consent for publication:** Not applicable.

**Availability of data and materials:** Not applicable.
