# Supplementary material for: Servicewomen’s experiences of managing pelvic health in occupational settings
Source: Womens Health (Lond). 2023 Jun 28;19:17455057231183839. doi: 10.1177/17455057231183839 (PMC10333990; doi:10.1177/17455057231183839)
Supplement: sj-docx-1-whe-10.1177_17455057231183839 – Supplemental material for Servicewomen’s experiences of managing pelvic health in occupational settings [file sj-docx-1-whe-10.1177_17455057231183839.docx]

Supplementary material

## Interview guide

**Introduction**

*Researcher introduction* - Thank you for your interest in our research, which is about women serving in the Australian Defence Force: an exploration of pelvic health issues. We are interested to find out about the pelvic health of female military members and how they manage it.

Please note, there are no right or wrong answers. I am interested in your perspectives and experiences.

*Review consent to participate* – Do you have any questions about the research or your participation?

You are free to say as much or as little as you want. If there are questions you don’t want to answer just let me know. The interview will take between 45 minutes to one hour but if you need to take a break please let me know. You are free to stop and withdraw from the interview at any time without any consequences or any questions asked.

*Are you happy for me to audio-record this interview?*

*Confirm consent to participate*

**General structure of Interview**

Questions will cover the following broad topic areas, and researchers will adapt the wording to the interviewee as required and follow relevant directions taken by the interviewee with follow-up questions in each area.

- Can you tell me about your experiences, in relation to pelvic health, that you’ve had during your time serving in the Australian Defence Force?
- Why do you think these experiences (positive or negative) occurred?
- Can you describe any ways that you feel these pelvic health experiences impacted your ability to do your job?
- You’ve described how these experiences impacted your physical ability to do your job. Did they impact you in any other ways?
- How do you feel these pelvic health experiences impacted your general health and wellbeing?
- What steps did you and/or the organisation take to ensure your pelvic health was managed to ensure your capacity to perform your work duties?
- You’ve mentioned how you and/or the organisation managed your pelvic health at work. Can you discuss if and how your strategies to manage your pelvic health have varied between your work and non-work life?
- Can you think of any ways in which the management of your pelvic health by you and/or the organisation could have been improved?
- Prompt interviewee to consider both self-management strategies and broader organisational strategies and supports.
